# Distinct Burnout Profiles in Physicians: Relationships with Occupational Satisfaction, Professional Identification, and Intellectual Humility

**DOI:** 10.3390/ijerph23080967

**Published:** 2026-07-26

**Authors:** Daniela Muntele-Hendreș, Georgiana-Mary Clapon, Clementina Doina Cojocaru

**Affiliations:** 1 Department of Psychology, Faculty of Psychology and Educational Sciences, “Alexandru Ioan Cuza” University, 700506 Iași, Romania; georgiana.clapon@uaic.ro; 2 Department of Medical Specialties, Faculty of Medicine, “Grigore T. Popa” University of Medicine and Pharmacy, 700115 Iași, Romania; doina.cojocaru@umfiasi.ro

**Keywords:** physician burnout, intellectual humility, professional social identification, job satisfaction, burnout profiles, healthcare professionals, cognitive dysfunction, occupational wellbeing, cluster analysis

## Abstract

**Highlights:**

**Public health relevance—How does this work relate to a public health issue?**

- Physician burnout represents an important occupational and public health issue associated with reduced wellbeing, impaired professional functioning, and potential risks for quality of patient care.
- This study examined burnout profiles among physicians and explored how professional identification and intellectual humility relate to occupational wellbeing in healthcare settings.

**Public health significance—Why is this work of significance to public health?**

- The findings support the heterogeneous nature of physician burnout, suggesting that physicians may experience distinct combinations of exhaustion, cognitive dysfunction, emotional dysfunction, and distancing.
- Professional identification and humility-related openness were associated with lower burnout-related dysfunction, highlighting potentially relevant psychological resources in demanding healthcare environments.

**Public health implications—What are the key implications or messages for practitioners, policymakers and/or researchers in public health?**

- Burnout prevention initiatives in healthcare settings may benefit from considering not only workload and organizational stressors, but also professional meaning, belonging, reflective functioning, and adaptive responses to uncertainty.
- Future research and intervention programs may further investigate whether intellectual humility and professional identification contribute to resilient and sustainable professional functioning among healthcare professionals.

**Abstract:**

Physician burnout represents a major occupational health concern associated with impaired wellbeing, reduced job satisfaction, and decreased quality of patient care. Although burnout is frequently examined using variable-centered approaches, less is known about distinct burnout profiles among physicians and their associations with professional identification and intellectual humility. The present study investigated relationships between burnout, job satisfaction, professional social identification, and intellectual humility among physicians using both variable-centered and person-centered approaches. A sample of 100 physicians completed measures assessing burnout dimensions, job satisfaction, professional identification, and intellectual humility. Cluster analysis identified four distinct burnout profiles characterized by different combinations of exhaustion, distancing, cognitive dysfunction, and emotional dysfunction. Significant differences between profiles emerged regarding job satisfaction and professional identification, with higher burnout generally associated with lower occupational wellbeing and weaker professional identification. Correlational analyses also indicated negative associations between burnout, job satisfaction, and professional identification. Although global intellectual humility was not significantly associated with burnout severity, exploratory analyses revealed that openness to correction and engagement in debate were negatively associated with cognitive and emotional dysfunction. These findings support the heterogeneous nature of physician burnout and suggest that professional identification and humility-related cognitive openness represent relevant psychological resources in demanding healthcare environments.

## 1. Introduction

Burnout is increasingly recognized as a multidimensional work-related syndrome that develops following prolonged exposure to occupational stress and is associated with substantial impairments in psychological functioning and professional performance. The Burnout Assessment Tool (BAT) operationalizes this multidimensional conceptualization by assessing four interrelated dimensions, exhaustion, mental distancing, cognitive impairment, and emotional impairment, thereby capturing both the core manifestations of burnout and their impact on cognitive, emotional, and occupational functioning [1]. Among physicians, burnout has become a major concern for healthcare systems and occupational health researchers because of its detrimental consequences for physicians’ well-being, healthcare organizations, and patient safety. Physicians are routinely exposed to persistent occupational stressors, including heavy workloads, emotional demands, time pressure, responsibility for high-stakes clinical decisions, and continuous interactions with patients, families, and multidisciplinary teams. Consistent evidence indicates that physician burnout is associated with reduced job satisfaction, impaired teamwork, poorer quality of patient care, increased medical errors, turnover intentions, and diminished psychological well-being [2,3]. These observations suggest that physicians may differ not only in the overall severity of burnout but also in the specific patterns of symptoms they experience, underscoring the value of approaches that acknowledge this heterogeneity and identify distinct burnout profiles.

To date, most research examining physician burnout has relied on variable-centered approaches, typically investigating associations between burnout and individual characteristics, workplace conditions, and occupational outcomes [2,3]. Although this body of research has substantially advanced our understanding of the factors associated with physician burnout, these approaches primarily estimate average relationships between variables across an entire sample and implicitly assume that the observed associations apply similarly to all individuals [4]. In contrast, person-centered approaches recognize that populations may comprise distinct subgroups of individuals characterized by different configurations of burnout symptoms rather than simply differing in their average level of burnout. Applied to physician burnout, this perspective acknowledges that individuals with comparable overall burnout scores may nevertheless experience different combinations of exhaustion, mental distancing, cognitive impairment, and emotional impairment. Identifying these distinct symptom configurations may reveal meaningful subgroups of physicians with different patterns of occupational functioning and vulnerability, thereby providing complementary insights into the heterogeneity of burnout that cannot be obtained from variable-centered analyses alone [4,5].

Accordingly, an increasing number of recent studies have adopted person-centered approaches to investigate burnout among healthcare professionals. Although these investigations have differed with respect to the professional groups examined, burnout measures employed, sample characteristics, and analytical techniques, they have consistently converged on the conclusion that burnout is a heterogeneous phenomenon comprising several distinct symptom patterns rather than representing a single continuum of severity [6,7,8]. Across studies, person-centered analyses have typically identified a low-burnout profile, a high-burnout profile, and one or more intermediate profiles characterized by different configurations of burnout symptoms. Although the exact number and composition of these profiles vary depending on the population studied and the assessment instrument employed, the findings consistently demonstrate that healthcare professionals with similar overall burnout levels may nevertheless differ substantially in the relative prominence and combination of burnout symptoms. This variability further supports the multidimensional nature of burnout and suggests that different symptom configurations may reflect distinct patterns of occupational adaptation rather than merely different levels of symptom severity. Furthermore, the identified profiles have been shown to differ not only in burnout severity but also in occupational well-being, emotional functioning, work engagement, professional adjustment, and other work-related outcomes, suggesting that they may represent groups with different support needs and responsiveness to preventive or organizational interventions [6,7,8]. Collectively, these findings indicate that person-centered approaches provide a richer understanding of burnout than global burnout scores alone by identifying meaningful subgroups of healthcare professionals with distinct occupational experiences and patterns of functioning. However, despite these advances, relatively little is known about the psychological and professional factors associated with different burnout profiles among physicians. Addressing this gap may contribute to a more comprehensive understanding of the factors that promote adaptive occupational functioning and resilience in demanding healthcare environments.

Professional social identification, reflecting the extent to which physicians identify with their professional group, has increasingly been recognized as an important factor that may shape how they perceive their professional role and respond to the demands of medical practice. It is closely related to the broader process of professional identity formation, through which physicians progressively develop a sense of themselves as members of the medical profession while internalizing its values, norms, and responsibilities [9]. Professional identity formation has been described as a continuous developmental process through which individuals come to think, act, and feel like physicians, integrating professional values into their self-concept and everyday clinical practice [10]. Within this framework, stronger professional social identification may foster a greater sense of belonging, professional commitment, and meaning at work, thereby influencing how physicians interpret occupational demands and cope with prolonged work-related stress. Empirical evidence supports this perspective. Jager et al. (2017) [11] reported that physicians experiencing higher levels of burnout were significantly less likely to perceive medicine as a calling, regard their work as meaningful, or indicate that they would choose the same profession again. Likewise, Monrouxe et al. (2017) [12] found that stronger professional identification among healthcare trainees was associated with lower burnout and psychological distress, suggesting that identification with the medical profession may represent an important psychological factor associated with physicians’ adaptation to demanding healthcare environments. Collectively, these findings suggest that professional social identification may contribute to explaining why physicians with similar levels of burnout differ in the ways they experience and respond to occupational demands.

Beyond professional social identification, intellectual humility represents another psychological characteristic that may facilitate adaptive functioning in demanding healthcare environments. Intellectual humility refers to the recognition that one’s knowledge, beliefs, and judgments are inherently limited and may require revision in light of new evidence or alternative perspectives [13]. Rather than reflecting low self-confidence or indecisiveness, intellectual humility is characterized by openness to learning, willingness to acknowledge personal limitations, and reduced defensiveness when confronted with uncertainty or disagreement. These characteristics are particularly relevant to medical practice, where physicians routinely face diagnostic uncertainty, rapidly evolving scientific evidence, and complex clinical decisions that require effective collaboration with colleagues and clear communication with patients. In such contexts, intellectual humility may facilitate reflective practice, openness to feedback, and more flexible responses to professional challenges. Building on these ideas, Michalec et al. (2024) [14] introduced the concept of professional humility, describing it as healthcare professionals’ capacity to recognize the limits of their own expertise while remaining receptive to the knowledge, perspectives, and contributions of others. According to the authors, cultivating professional humility may strengthen collaboration, patient-centered care, and lifelong learning within healthcare teams. However, despite growing theoretical interest in humility-related constructs, empirical research examining the role of intellectual humility in physician burnout remains scarce. Consequently, it is still unclear whether intellectual humility contributes to explaining why physicians with similar levels of burnout may nevertheless differ in their psychological functioning and adaptation to occupational demands.

Taken together, the available evidence suggests that physician burnout is a multidimensional and heterogeneous phenomenon that cannot be fully understood through variable-centered approaches alone [4,5]. Although recent person-centered studies have advanced our understanding of distinct burnout profiles [6,7,8], relatively little is known about the psychological and professional factors associated with these profiles, particularly among physicians. Furthermore, professional social identification and intellectual humility have largely been investigated separately, and their potential contribution to explaining differences in burnout experiences remains insufficiently understood. Addressing these gaps may contribute to a more comprehensive understanding of physicians’ adaptation to occupational demands and help identify factors that may inform future prevention and intervention strategies.

Against this background, the present study examined the relationships among burnout, job satisfaction, professional social identification, and intellectual humility in a sample of physicians using a person-centered approach. Specifically, we first identified distinct burnout profiles based on the four dimensions assessed by the Burnout Assessment Tool. We then examined whether these profiles differed with respect to job satisfaction, professional social identification, and intellectual humility. Finally, we investigated whether professional social identification moderated the association between job satisfaction and burnout and conducted exploratory analyses examining the associations between specific dimensions of intellectual humility and individual dimensions of burnout.

## 2. Materials and Methods

### 2.1. Participants and Procedure

The study employed a cross-sectional design to investigate the relationships between burnout, job satisfaction, professional social identification, and intellectual humility among physicians. In addition to variable-centered analyses, person-centered burnout profiles were explored using cluster analysis.

The final sample consisted of 100 licensed physicians practicing in Romania, recruited using convenience sampling. The invitation to participate was distributed online by one of the co-authors, a practicing physician, through professional online communities and networks of physicians. Participants ranged in age from 25 to 76 years (M = 38.88, SD = 10.44), and their length of professional experience ranged from 1 to 45 years (M = 12.57, SD = 9.66). The sample comprised 82 women (82%) and 18 men (18%). Regarding marital status, 66% of participants were married, 20% were in a committed relationship, 10% were single, and 4% were divorced. Participation was voluntary and anonymous. Before completing the online questionnaire administered via Google Forms, participants were informed about the purpose of the study, the confidential handling of their responses, and their right to withdraw at any time before submitting the survey. Only physicians who provided informed consent were included in the study.

### 2.2. Measures

#### 2.2.1. Burnout

Burnout was assessed using the Burnout Assessment Tool (BAT) [1,15], a 23-item self-report instrument designed to assess the core symptoms of burnout across four dimensions: exhaustion (8 items), mental distance (5 items), cognitive impairment (5 items), and emotional impairment (5 items). The instrument was translated into Romanian using a forward–backward translation procedure to ensure semantic equivalence with the original version. An example item is: “At work, I get tired very quickly.” Participants responded using a five-point Likert scale ranging from 1 (never) to 5 (always), with higher scores indicating greater burnout severity. Mean scores were calculated for each dimension. Internal consistency coefficients in the present sample were satisfactory to excellent: exhaustion (α = 0.890), mental distance (α = 0.775), cognitive impairment (α = 0.908), and emotional impairment (α = 0.888). The four burnout dimensions were used in the cluster analysis, whereas the overall burnout score was used in the moderation analyses.

#### 2.2.2. Job Satisfaction

Job satisfaction was assessed using the Work Satisfaction among Physicians Scale developed by Bovier and Perneger [16], a 17-item self-report instrument designed to assess physicians’ satisfaction with multiple aspects of their professional life, including relationships with patients and colleagues, workload, income, professional autonomy, opportunities for continuing education, and overall job satisfaction. An example item is: “The quality of care you are able to provide.” The instrument was translated into Romanian using a forward–backward translation procedure to ensure semantic equivalence with the original version. Participants responded on a seven-point Likert scale ranging from 1 (very dissatisfied) to 7 (extremely satisfied), with higher scores indicating greater job satisfaction. Internal consistency coefficients ranged from α = 0.729 to α = 0.857 across the instrument dimensions. Higher scores reflected higher occupational satisfaction.

#### 2.2.3. Professional Social Identification

Professional social identification was conceptualized as one component of physicians’ broader professional identity, reflecting the extent to which individuals identify with and feel attached to their professional group. It was assessed using the Social Identity Questionnaire developed by Roccas and Klar [17], a 16-item self-report instrument. The instrument was translated into Romanian using a forward–backward translation procedure to ensure semantic equivalence with the original version. Participants responded on a six-point Likert scale ranging from 1 (strongly disagree) to 6 (strongly agree), with higher scores indicating stronger professional social identification. An example item is “Being a member of the group of physicians is an important part of my identity.” Although the instrument comprises two dimensions (attachment to the professional group and pride associated with group membership), the total score was used in the present study as an overall indicator of professional social identification. Internal consistency coefficients for the two dimensions were good (attachment to the group: α = 0.859; pride in group belonging: α = 0.845). The overall scale also demonstrated excellent internal consistency (α = 0.915).

#### 2.2.4. Intellectual Humility

Intellectual humility was assessed using the Multidimensional Intellectual Humility Scale developed by Alfano et al. [18], a 22-item multidimensional self-report instrument. For the purposes of the present study, analyses focused on three dimensions of the instrument: modesty, willingness to be corrected, and openness to debate. The instrument was translated into Romanian using a forward–backward translation procedure to ensure semantic equivalence with the original version. Participants responded on a seven-point Likert scale ranging from 1 (strongly disagree) to 7 (strongly agree), with higher scores indicating higher levels of intellectual humility. An example item is: “I appreciate being corrected when I am wrong.” Internal consistency coefficients in the present sample were acceptable: modesty (α = 0.734, after removal of Item 7), willingness to be corrected (α = 0.762), and openness to debate (α = 0.747). Both the overall intellectual humility score and the individual dimensions were examined in the present study.

#### 2.2.5. Statistical Analysis

Data analyses were conducted using Jamovi statistical software (version 2.6.44). Descriptive statistics and Pearson correlations were first computed for the main study variables. Subsequently, a k-means cluster analysis based on the four burnout dimensions was conducted to identify distinct burnout profiles among physicians. The four-cluster solution was retained based on its interpretability, conceptual meaningfulness, and adequate cluster sizes. Variables were not standardized prior to the k-means cluster analysis because all four burnout dimensions were derived from the same instrument and measured on the same response scale, allowing them to contribute equally to the clustering procedure. Differences between burnout profiles were examined using one-way analyses of variance (ANOVA). In addition, a moderation analysis based on linear regression was conducted to examine whether professional social identification moderated the relationship between job satisfaction and burnout. Finally, exploratory correlational analyses examined associations between theoretically relevant dimensions of intellectual humility and burnout-related cognitive and emotional dysfunction. Clustering was performed using the Hartigan–Wong k-means algorithm based on Euclidean distance. To improve the stability of the clustering solution, the analysis was initialized using 10 random starting values, and the best-performing solution was retained.

## 3. Results

### 3.1. Descriptive Statistics and Correlations

Preliminary analyses were conducted to examine whether demographic characteristics were associated with the primary study variables. Female physicians reported slightly higher levels of exhaustion than male physicians, t(98) = −2.02, *p* = 0.046, whereas no significant gender differences were observed for overall burnout, mental distance, cognitive impairment, emotional impairment, job satisfaction, professional identification, or intellectual humility. Age was not significantly associated with any of the study variables. Years of professional practice showed a weak negative correlation with cognitive impairment (r = −0.24, *p* = 0.016), but was not significantly associated with the remaining study variables. In addition, cognitive impairment differed according to marital status, F(3,96) = 3.43, *p* = 0.020, whereas no significant differences according to marital status were observed for overall burnout, the remaining burnout dimensions, job satisfaction, professional identification, or intellectual humility.

Descriptive statistics and Pearson correlations for the main study variables are presented in Table 1. Overall, the sample was characterized by moderate levels of burnout, relatively high job satisfaction, moderate-to-high professional social identification, and relatively high intellectual humility.

Burnout was strongly and negatively associated with job satisfaction.

### 3.2. Burnout Profiles

To further investigate the heterogeneity of burnout manifestations among physicians, a k-means cluster analysis was conducted using the four burnout dimensions: exhaustion, mental distancing, cognitive dysfunction, and emotional dysfunction. Several cluster solutions were explored before selecting the final model. The four-cluster solution was retained because it provided the best balance between interpretability, conceptual meaningfulness, and adequate cluster sizes. Solutions with fewer clusters merged conceptually distinct burnout profiles, whereas solutions with more clusters yielded smaller clusters that were more difficult to interpret meaningfully. Given the exploratory nature of the study, cluster analysis was used to identify potentially meaningful burnout profiles that should be confirmed in future studies with larger samples.

The first cluster (*n* = 28) was characterized by elevated exhaustion levels combined with comparatively moderate levels of distancing and dysfunction and was labeled the Exhaustion-Dominant Profile. The second cluster (*n* = 20) displayed the highest scores across all burnout dimensions and was labeled the High-Burnout Profile. The third cluster (*n* = 18) showed the lowest scores on all burnout dimensions and was labeled the Low-Burnout Profile. Finally, the fourth cluster (*n* = 34) presented intermediate burnout levels and was labeled the Moderate-Burnout Profile. Overall, the findings suggest that burnout among physicians is heterogeneous and may manifest in distinct psychological configurations rather than as a unitary construct.

As shown in Table 2, the four cluster centers displayed clearly differentiated patterns across the four burnout dimensions, supporting the distinctiveness of the identified burnout profiles.

### 3.3. Differences Between Burnout Profiles

One-way ANOVA analyses revealed significant differences between burnout profiles in terms of job satisfaction, F(3,96) = 16.16, *p* < 0.001, η^2^ = 0.34, and professional social identification, F(3,96) = 4.64, *p* = 0.004, η^2^ = 0.13. No significant differences emerged for intellectual humility, F(3,96) = 1.66, *p* = 0.180, η^2^ = 0.05. As shown in Table 3, physicians in the High-Burnout Profile reported the lowest levels of job satisfaction and professional social identification, whereas those in the Low-Burnout and Moderate-Burnout Profiles reported the highest levels. The Exhaustion-Dominant Profile was characterized by elevated exhaustion while maintaining intermediate levels of job satisfaction and professional social identification, suggesting the possibility of an earlier or less generalized stage of burnout.

### 3.4. Moderation Analysis

To examine whether professional social identification moderated the association between job satisfaction and burnout, a linear regression moderation analysis was conducted (Table 4). Job satisfaction was a significant negative predictor of burnout (β = −1.237, *p* < 0.001), whereas professional social identification was not significantly associated with burnout (β = −0.829, *p* = 0.057). The interaction between job satisfaction and professional social identification approached, but did not reach, statistical significance (β = 0.010, *p* = 0.051). Therefore, the hypothesized moderation effect was not supported. Nevertheless, the near-significant interaction suggests that professional social identification may attenuate the association between reduced job satisfaction and burnout, a possibility that should be examined in future studies with larger samples.

### 3.5. Exploratory Analyses on Intellectual Humility Dimensions

Because total intellectual humility scores were not significantly associated with overall burnout levels, exploratory analyses were conducted to examine whether specific dimensions of intellectual humility were differentially associated with burnout dimensions. Modesty was not significantly associated with any burnout dimension. In contrast, willingness to be corrected was negatively associated with cognitive dysfunction (r = −0.269, *p* = 0.007) and emotional dysfunction (r = −0.209, *p* = 0.037), whereas openness to debate was negatively associated with cognitive dysfunction (r = −0.210, *p* = 0.036). No other significant associations were observed. These exploratory findings suggest that specific facets of intellectual humility, particularly willingness to be corrected and openness to debate, may be associated with lower levels of burnout-related cognitive and emotional impairment.

## 4. Discussion

The present study explored the relationships between burnout, professional identification, job satisfaction, and intellectual humility among physicians, while also examining whether distinct burnout profiles could be identified within the sample. Several important findings emerged. First, burnout was negatively associated with both job satisfaction and professional identification. Second, cluster analysis suggested the existence of multiple burnout profiles characterized by different levels of exhaustion, distancing, cognitive dysfunction, and emotional dysfunction. Third, exploratory analyses indicated that specific dimensions of intellectual humility, particularly willingness to be corrected and openness to debate, were modestly associated with lower levels of cognitive and emotional dysfunction. Finally, professional identification did not significantly moderate the relationship between job satisfaction and burnout, although the interaction term approached statistical significance.

The identification of distinct burnout profiles is consistent with recent person-centered approaches to burnout research, which suggest that burnout should not be conceptualized as a homogeneous syndrome. Studies conducted among healthcare professionals have repeatedly shown that clinicians may experience different combinations of exhaustion, disengagement, inefficacy, and cognitive symptoms rather than a single uniform pattern. Haire et al. (2024) [6], for example, identified several latent burnout profiles among hospital doctors, including engaged, overextended, disengaged, ineffective, and severe burnout profiles, emphasizing the value of profile-based approaches for understanding physician wellbeing. Similarly, Ridremont and Boujut (2025) [7] reported both endpoint and intermediate burnout profiles among pediatric healthcare professionals, highlighting the heterogeneity of burnout experiences and the practical importance of tailored interventions. Comparable findings have also been observed among medical residents, where cluster analytic approaches identified markedly different patterns of wellbeing, professional engagement, and emotional distress [8].

Taken together, these findings strengthen the argument that physician burnout is multidimensional and may manifest differently across individuals. The current results extend this literature by suggesting that burnout profiles can also be differentiated using dimensions included in the Burnout Assessment Tool, particularly cognitive and emotional dysfunction. This distinction may be clinically important because cognitive symptoms often receive less attention in burnout research despite their substantial implications for medical decision-making, concentration, memory, flexibility, and patient safety.

A systematic review and meta-analysis by Gavelin et al. (2022) [19] concluded that clinical burnout is associated with impairments across several cognitive domains, including executive functioning, attention, working memory, processing speed, and episodic memory. Similarly, Koutsimani and Montgomery (2022) [20] argued that burnout-related cognitive difficulties may be underestimated in healthcare settings, especially regarding executive control and visuospatial functioning. In highly demanding medical environments, where rapid decisions, sustained attention, and flexible reasoning are essential, such impairments may affect not only physicians’ wellbeing but also professional functioning and quality of care.

An additional important finding concerns the role of professional identification. Higher professional identification was associated with lower burnout, supporting previous evidence suggesting that identification with one’s professional role may function as a psychological resource in stressful healthcare environments. Earlier studies have shown that stronger professional identity is associated with lower burnout, higher wellbeing, and better professional adjustment among healthcare professionals [21,22]. Similar patterns have also been observed among medical students, where stronger professional identity was related to lower emotional exhaustion and cynicism [23]. In the present study, physicians who reported stronger identification with their professional group also tended to report lower burnout severity, suggesting that belongingness, professional meaning, and shared professional values may contribute to occupational resilience.

Although the moderation effect did not reach statistical significance, the near-significant interaction may reflect a potentially meaningful pattern that warrants further investigation in larger samples. Professional identification may contribute to physicians’ sense of meaning, continuity, and belonging in demanding healthcare environments. At the same time, previous research suggests that strong professional identification may also involve emotional costs under certain circumstances. For example, Caricati et al. (2021) [24] found that professional identification was associated with lower burnout but greater vulnerability to secondary traumatic stress under stigmatizing conditions. These findings highlight the complex role of professional identification as a potential psychological resource whose effects may depend on contextual factors.

Although the overall intellectual humility score was not significantly associated with burnout, exploratory analyses revealed that specific dimensions of intellectual humility were modestly associated with cognitive and emotional dysfunction. In particular, willingness to be corrected and openness to debate were associated with lower levels of cognitive and emotional impairment. These findings suggest that intellectual humility may not function as a unitary protective factor against burnout, but that certain facets of the construct may be more closely related to adaptive cognitive and emotional responses in demanding professional contexts.

This interpretation is consistent with contemporary perspectives that conceptualize intellectual humility as an adaptive disposition characterized by openness to new information, awareness of personal limitations, and willingness to revise one’s views in light of new evidence. In healthcare settings, these characteristics may facilitate effective collaboration, reflective practice, and adaptation to the uncertainty that is inherent in clinical decision-making. Previous research has suggested that humility may support teamwork, openness to feedback, and professional learning while helping clinicians navigate complex and ambiguous situations without becoming overly rigid or defensive [25,26,27,28,29].

These perspectives are particularly relevant for interpreting the present findings regarding cognitive and emotional dysfunction. It is possible that intellectually humble physicians may experience lower burnout-related cognitive impairment because humility promotes greater flexibility in processing uncertainty, feedback, disagreement, and complex professional situations. Rather than responding defensively to ambiguity or interpersonal challenge, intellectually humble individuals may demonstrate greater willingness to seek assistance, revise interpretations, tolerate uncertainty, and collaboratively solve problems. Such processes may help reduce cognitive rigidity, emotional overload, and maladaptive perfectionistic tendencies frequently associated with burnout. At the same time, alternative explanations should also be considered. Because the study employed a cross-sectional design, physicians experiencing lower levels of cognitive and emotional dysfunction may be more willing to acknowledge personal limitations, accept corrective feedback, and engage in constructive dialogue. In addition, the observed associations may reflect broader individual characteristics, such as psychological flexibility or adaptive coping styles, rather than the specific effects of intellectual humility itself. Furthermore, previous research has suggested that women and men may differ in intellectual humility and related interpersonal characteristics, although findings have not been entirely consistent across studies. For example, Mukhtar et al. (2023) [30] reported higher levels of intellectual humility among men, whereas Buljan Šiber et al. (2023) [31] found that female managers were perceived by their subordinates as displaying greater expressed intellectual humility. These contrasting findings suggest that gender differences may depend on the conceptualization and assessment of intellectual humility. Future longitudinal studies with larger and more diverse samples are needed to clarify these alternative explanations and to examine whether gender influences these relationships.

Several limitations should nevertheless be acknowledged. First, the cross-sectional design precludes causal conclusions regarding the relationships among burnout, job satisfaction, professional identification, and intellectual humility. Second, all variables were assessed using self-report measures, which may increase shared method variance and social desirability bias. Third, the sample size, although adequate for the exploratory analyses conducted in the present study, limits the stability and generalizability of the identified burnout profiles and the statistical power to detect interaction effects in the moderation analysis. Replication in larger and more diverse samples of physicians is therefore warranted. In addition, information regarding participants’ parenting status was not collected. Because family responsibilities may influence work-related stress and burnout among physicians, future studies should consider including this variable to provide a more comprehensive understanding of factors associated with burnout. Another limitation concerns the predominance of women in our sample (82%). Although this distribution broadly reflects the composition of the medical workforce, the overrepresentation of women may have influenced the observed findings and may limit their generalizability. Future studies should aim to recruit more gender-balanced samples and further examine potential gender differences in burnout and its associated psychological correlates. Finally, the exploratory analyses concerning intellectual humility should be interpreted with caution until replicated in future studies specifically designed to investigate humility-related processes in healthcare professionals.

Despite these limitations, the present study makes several contributions to the literature on physician burnout. First, it demonstrates the heterogeneity of burnout by identifying distinct burnout profiles based on the Burnout Assessment Tool dimensions, including cognitive and emotional dysfunction. Second, it highlights the importance of professional identification as a factor associated with lower burnout levels. Finally, it provides preliminary evidence suggesting that specific dimensions of intellectual humility, rather than intellectual humility as a global construct, may be related to burnout-related cognitive and emotional functioning. By integrating person-centered and variable-centered approaches, the findings contribute to a more nuanced understanding of physician burnout and may inform the development of interventions that address not only occupational demands but also professional identity and adaptive psychological resources.

## 5. Conclusions

The present study contributes to the growing literature emphasizing the complex and heterogeneous nature of physician burnout. By combining person-centered analyses with the investigation of professional identification and intellectual humility, the findings suggest that burnout among physicians is associated not only with occupational strain, but also with cognitive, interpersonal, and identity-related processes.

The results indicate that physicians may present distinct burnout profiles characterized by different combinations of exhaustion, distancing, cognitive dysfunction, and emotional dysfunction. In addition, lower burnout was associated with higher job satisfaction and stronger professional identification, supporting previous evidence regarding the protective role of professional meaning and occupational belonging in healthcare settings.

Although intellectual humility was not globally associated with burnout severity, some humility-related dimensions, particularly openness to correction and willingness to engage in dialogue, were linked to lower cognitive and emotional dysfunction. These findings suggest that humility-related characteristics may contribute to more adaptive responses to professional stress, especially in medical environments characterized by uncertainty, complexity, and continuous interpersonal demands.

More broadly, the findings support emerging perspectives suggesting that effective professional functioning in healthcare may depend not only on technical competence and resilience, but also on reflective openness, tolerance for uncertainty, cognitive flexibility, and collaborative engagement. From this perspective, intellectual humility may represent a potentially relevant, although still insufficiently explored, psychological resource in physicians’ occupational wellbeing.

Despite the exploratory nature of some analyses and the limitations associated with the cross-sectional design and relatively modest sample size, the present study highlights several promising directions for future research. Further longitudinal and larger-scale studies could clarify how burnout profiles develop over time and whether professional identification and humility-related characteristics may contribute to resilience, adaptive functioning, and sustainable professional wellbeing among healthcare professionals.

## Figures and Tables

**Table 1:** Means, Standard Deviations, and Correlations Between Main Study Variables.

Variable	M	SD	1	2	3	4
1. Burnout total	56.2	13.5	-			
2. Job satisfaction total	78.1	15.0	−0.622 ***	-		
3. Professional social identification	65.4	14.5	−0.372 ***	0.598 ***	-	
4. Intellectual humility total	113.0	16.6	−0.189	−0.007	−0.056	-

Note. *** *p* < 0.001.

**Table 2:** Mean Burnout Dimension Scores Across the Four Burnout Profiles.

Burnout Dimensions	Exhaustion-Dominant Profile (*n* = 28)	High-Burnout Profile (*n* = 20)	Low-Burnout Profile (*n* = 18)	Moderate-Burnout Profile (*n* = 34)
Exhaustion	28.04	29.25	19.44	20.79
Mental distancing	11.54	15.25	7.17	9.06
Cognitive dysfunction	10.93	15.90	5.56	10.47
Emotional dysfunction	10.82	15.50	6.39	9.47

Note. Values represent mean scores for each burnout dimension within each burnout profile.

**Table 3:** Descriptive Statistics Across Burnout Profiles.

Variable	Exhaustion-Dominant Profile	High-Burnout Profile	Low-Burnout Profile	Moderate-Burnout Profile
Job satisfaction total	74.3 (11.4)	63.9 (13.7)	86.6 (13.6)	85.2 (11.8)
Professional social identification	66.1 (14.1)	55.4 (14.6)	68.9 (14.5)	68.8 (12.6)
Intellectual humility total	113.3 (14.4)	107.4 (21.5)	119.3 (13.6)	113.7 (16.1)

Note. Values represent means with standard deviations in parentheses.

**Table 4:** Moderation Analysis Predicting Burnout.

Predictor	B	SE	t	*p*
Intercept	152.59	27.25	5.60	<0.001
Job satisfaction	−1.237	0.354	−3.50	<0.001
Professional social identification	−0.829	0.430	−1.93	0.057
Job Satisfaction × Professional Identification	0.010	0.005	1.97	0.051

Note. R = 0.641, R^2^ = 0.410.

## Data Availability

The data presented in this study are available from the corresponding author upon reasonable request.
